# Social interactions offset the detrimental effects of digital media use on children’s vocabulary

**DOI:** 10.3389/fdpys.2024.1401736

**Published:** 2024-05-27

**Authors:** Sarah C. Kucker, Julie M. Schneider

**Affiliations:** 1 Department of Psychology, Southern Methodist University, Dallas, TX, United States; 2 Department of Communication Science and Disorders, Louisiana State University, Baton Rouge, LA, United States

**Keywords:** digital media, social interactions, vocabulary, socioeconomic status, language development

## Abstract

Young children’s rapid vocabulary growth during the first few years is supported by input during social interactions with caregivers and, increasingly, from digital media. However, the amount of exposure to both sources can vary substantially across socioeconomic classes, and little is known about how social interactions and digital media use together predict vocabulary in the first few years of life. The current study takes a first step toward examining whether increased social interactions with other individuals may buffer the potentially detrimental effects of digital media use on language among a socioeconomically diverse sample. 305 caregivers of children between 17 and 30-months completed questionnaires about their family demographics, their child’s technology use, and the child’s daily routines and social interactions. Findings suggest children who experience fewer human interactions and greater technology exposure have smaller vocabularies than their peers who socialize more and use less technology, and this disparity becomes greater as children get older. Moreover, the number of social interactions moderates the link between SES, digital media, and vocabulary such that the negative impact of digital media on vocabulary for children from low SES households can be offset with increased social interactions. Together, this suggests that increasing the amount of human interactions may serve as a protective factor for vocabulary outcomes in a world where digital media use is prominent.

## 1 Introduction

During the first years of life, young children’s vocabulary expands rapidly, from 50 words at 18-months to over 500 by 30-months (Fenson et al., 1994). This rapid growth is fueled, in part, by relevant language input from, and interactions with, social partners (Hoff, 2006; Rowe, 2008). Such interactions are beneficial for multiple reasons – not only do they provide linguistic input, but they also give children opportunities for dyadic conversations and exposure to pragmatic elements supporting language growth. Problematically though, distractions and interruptions in children’s environments associated with the use of digital media can reduce both the quantity and quality of language input (Reed et al., 2017) and subsequent vocabulary growth (Madigan et al., 2020). It is especially important that we characterize such distractions and interruptions in the early language environments of children from lower socioeconomic status (SES) households, as they are shown to experience less vocabulary growth compared to their higher SES peers (Hart and Risley, 1995; Golinkoff et al., 2019). Notably, early evidence suggests that disruptions from digital media may be more pronounced for children from low SES households, having downstream negative effects on their language development (Dynia et al., 2021). However, prior work has suggested that interacting with multiple communicative partners can improve communication skills (Lev-Ari and Sebanz, 2020), suggesting that social interactions could offset the negative association between digital media use and vocabulary. In the current study, we examine whether increased social interactions broadly (including those outside the home) may buffer the potentially detrimental vocabulary effects of digital media use among a socioeconomically diverse sample.

### 1.1 The rise of digital media

By the time children are 2 years old, they experience nearly 2 h of screen time per day (Kucker et al., 2024); an amount that rises as children age. More media use by young children is associated with a smaller vocabulary size (Madigan et al., 2020). This is particularly true when children engage in solo, passive video viewing without a caregiver or social partner (Lytle et al., 2018). The general consensus is that while there are beneficial uses of digital media (e.g., educational, social connection, joint engagement Linebarger and Vaala, 2010; Lytle et al., 2018), the omniprescence of digital media in young children’s lives has the potential to hinder language development. One primary reason for this is that heightened media exposure can diminish and replace the rich social interactions known to foster language growth. For example, higher rates of digital media use predict fewer child-directed utterances (Pempek et al., 2014; Lederer et al., 2022), fewer conversational turns between children and caregivers (Cycyk and De Anda, 2021; Sundqvist et al., 2021), and less vocalization by the child (Ferjan Ramírez et al., 2021).

Digital media use is also significantly more prevalent in lower SES households (Rideout and Robb, 2020; Dore and Dynia, 2021). In particular, TV consumption is higher among lower SES, Black families (Yang-Huang et al., 2017; Stoll, 2023) who report significantly higher use of background TV, especially for infants (Lapierre et al., 2012). These differences in how families use background TV may have unique downstream impacts on language input (Skoe et al., 2013) wherein increased exposure to background TV can either promote or hinder children’s ability to learn new words, impacting vocabulary growth. In fact, research has shown that higher rates of digital media exposure in lower SES households is associated with lower expressive language skills (Dynia et al., 2021). Together, digital media use appears to diminish opportunities for face-to-face social interactions and opportunities for children to use their growing language skills, and this may be particularly troublesome for lower SES families.

### 1.2 The importance of social interactions

Social interactions are arguably one of the most important mechanisms supporting children’s language growth. Interactions with both adults and children bring opportunities for hearing language input, practicing talking themselves, and engaging in language-relevant pragmatic behaviors, all of which support language growth (Hoff, 2006). For instance, more social contacts at the start of preschool predict increases in verbal and non-verbal language skills by the end of the year (Hofmann and Müller, 2021), and the more preschoolers interact with their peers, the more likely they are to talk to both their peers and teachers, in turn predicting vocabulary growth (Perry et al., 2018). Social interactions in the home are also critical (e.g. Ramírez-Esparza et al., 2014), as numerous studies have shown that the quantity and quality of language input from caregivers promotes language development (Hart and Risley, 1995; Rowe, 2012; Romeo et al., 2018) and too much background noise or chaos might diminish language (Lecheile et al., 2020). As children get older, their social interactions become increasingly mature and important for continued language growth (Ramírez-Esparza et al., 2017).

However, the quantity and quality of these social interactions varies widely. Classic studies of children from lower SES households have argued that children hear less child-directed speech (Schwab and Lew-Williams, 2016) and less language input from caregivers (Rowe, 2012). However, other work with low SES households has indicated language input often comes from a variety of other communicative partners in these households (Shneidman and Goldin-Meadow, 2012; Shneidman et al., 2013; Sperry et al., 2019; referred to often as overheard speech). When other speakers beyond the primary caregiver are accounted for, differences in input are often diminished (Sperry et al., 2019; Dailey and Bergelson, 2022). This means that broader opportunities for social interactions beyond just the primary caregiver are likely critical, especially for diverse samples and it is important to consider the role of other individuals in children’s environments, beyond primary caregivers and home-based interactions. Doing so can help us develop a more holistic understanding of the relationship between daily social interactions and language development (Poudel et al., 2024). It also means that the facilitatory role of multiple communicative partners in promoting children’s language development is especially relevant for children who come from diverse socioeconomic households.

### 1.3 Current study

Taken together, both the amount of time a child spends engaging with digital media, and their opportunities for social interactions broadly, impact their developing vocabulary knowledge. Indeed, children exposed to more media hear less child-directed speech (Christakis et al., 2009; Anderson and Hanson, 2017), and higher rates of media use in the home (primarily by caregivers) result in less dyadic turn-taking and conversations with children (Sundqvist et al., 2021). Reductions in language input associated with higher rates of digital media use have negative downstream effects on vocabulary size. However, patterns of digital media use and social interactions vary as children get older and across SES groups, with children from lower SES household being exposed to more media, having more communicative partners, yet still being at risk for language delays. Given interactions with multiple social partners can reduce digital media use and increase language input, the amount of social partners a child has may offset the link between media use and vocabulary. However, a direct test of the relationship between children’s own media use, overall number of social interactions, and vocabulary, especially in a diverse sample, has not been assessed. Because of this, the pathways by which media use alters language development remain unknown.

## 2 Materials and methods

### 2.1 Participants

Caregivers of children 17–30-months-old were recruited to participate online through Cloud Research between February 2022 and April 2023. All completed surveys were screened for inattentive/illegitimate responses and data were cleaned according to guidelines for online data collection (Chmielewski and Kucker, 2020). Specifically, responses that had inconsistency in reporting their child’s birthday, irregular free response answers, repeated submission of the surveys, or were ineligible due to being outside the age range or not being exposed to English were not included in the final sample (*n* = 103). The final sample included 305 caregivers (*n*_female_ = 209) of 17–30-month-old children (*n*_female_ = 135) from a wide variety of socioeconomic backgrounds *(M*_income_ $75,000, Range: <$10,000 to >$200,000; *M*_education_ 2-year college degree, Range: 8th grade – Doctoral degree), but were largely White (Caregiver: 81%; Child: 75%) and non-Hispanic (Caregiver: 92.5%; Child: 89%) (see Supplementary Table S1 for full demographic information).

Using the pwr.f2.test function from the pwr package (Cohen, 1988) of R (R Core Team, 2020), we calculated the sample size necessary to execute multiple regression analyses with 4 predictor variables. The Cohen.ES function verified that a value of 0.15 represented the ability to detect medium effect sizes (Cohen, 1988). Using these medium effect sizes, with a significance level at 0.05, and power at 90%, we calculated the sample size necessary to execute our analysis at 103. We also used the ssMediation.VSMc function from the powerMediation package (Vittinghoff et al., 2009) of R to compute the sample size needed to reliably conduct a mediation analysis. Using the same power and effect size stated above, with the regression coeffcient for the mediator set at 0.04, we calculated the sample size necessary to execute our analysis was 118. Therefore, we have suffcient power to conduct all subsequent analyses.

### 2.2 Materials and procedure

Caregivers completed questionnaires about family demographics (parent education, income, employment status, ethnicity, race), and their child’s digital media use. Because prior work has found that the majority of children’s digital media time at this age is spent with videos/TV and most children have some level of regular TV time (Kucker et al., 2024), the average minutes/day spent watching videos/TV/movies from the Media Assessment Questionnaire (MAQ; Barr et al., 2020) was used as the metric for digital media use. Children’s expressive vocabulary was measured with the MacArthur-Bates Communicative Development Inventory: Words and Sentences (MCDI; Fenson et al., 1994). Children’s total amount of social interactions was assessed through a self-report asking “On average, how many people does your child interact with on a daily basis?”^1^. The study was approved by the Oklahoma State University and Southern Methodist University Internal Review Boards and all participants gave informed consent.

### 2.3 Analysis

The goal of the current analysis is to pinpoint how digital media and social interactions influence children’s vocabulary learning. Given each of these variables differ across development and vary on the basis of SES, we further probed how age and SES differentially impact the relationship between digital media, social interactions and vocabulary. First, we include bivariate correlations between all variables of interest. We next evaluated how the association between digital media and social interactions varies across ages by utilizing a multiple regression model with a three-way interaction between these terms and vocabulary as the outcome variable. In this model, age is used as a possible moderator by which the impact of media and social interactions change as children get older, while controlling for SES. Given SES-based differences in both vocabulary and digital media use are highly reported, we next used a serial mediation model to identify whether digital media use is the process by which vocabulary differences exist across SES. Lastly, to identify whether the number of social interactions children engage in can offset SES-based differences in vocabulary and digital media use, we ran conditional processes (i.e., moderated-mediation), by including social interactions as a moderator in the above mediation model.

## 3 Results

### 3.1 Bivariate correlations

Children’s average vocabulary size (based on the MCDI) was 175.90 words (*SD* = 173.57, Range: 0–664), their average daily digital media use was 122.39 min/day (*SD* = 103.18, Range: 0–480), and they engage with an average of 5.54 people/day (*SD* = 4.27, Range: 1–30). As children got older, they also increased their number of social interactions [*r*(297) = 0.12*, p* = 0.04]. Consistent with prior work, Pearson’s correlations revealed greater digital media use (TV/video time) was associated with less vocabulary knowledge [*r*(305) = −0.13, *p* = 0.03]. More digital media use was also associated with lower rates of social interaction, *r*(297) = −0.21, *p* < 0.001, coming from a household with lower rates of parental education, *r*(305) = 0.14, *p* = 0.01, and less income [*r*(303) = 0.13, *p* = 0.02]. Given average parental education and income held similar relationships with other variables of interest, and are often combined in studies of SES, all subsequent analyses utilized a composite measure of SES, wherein the rank order of average parental education and income were averaged together. All results are included in Table 1.

### 3.2 At what age do social interactions offset the relationship between digital media use and vocabulary?

**FIGURE 1:**
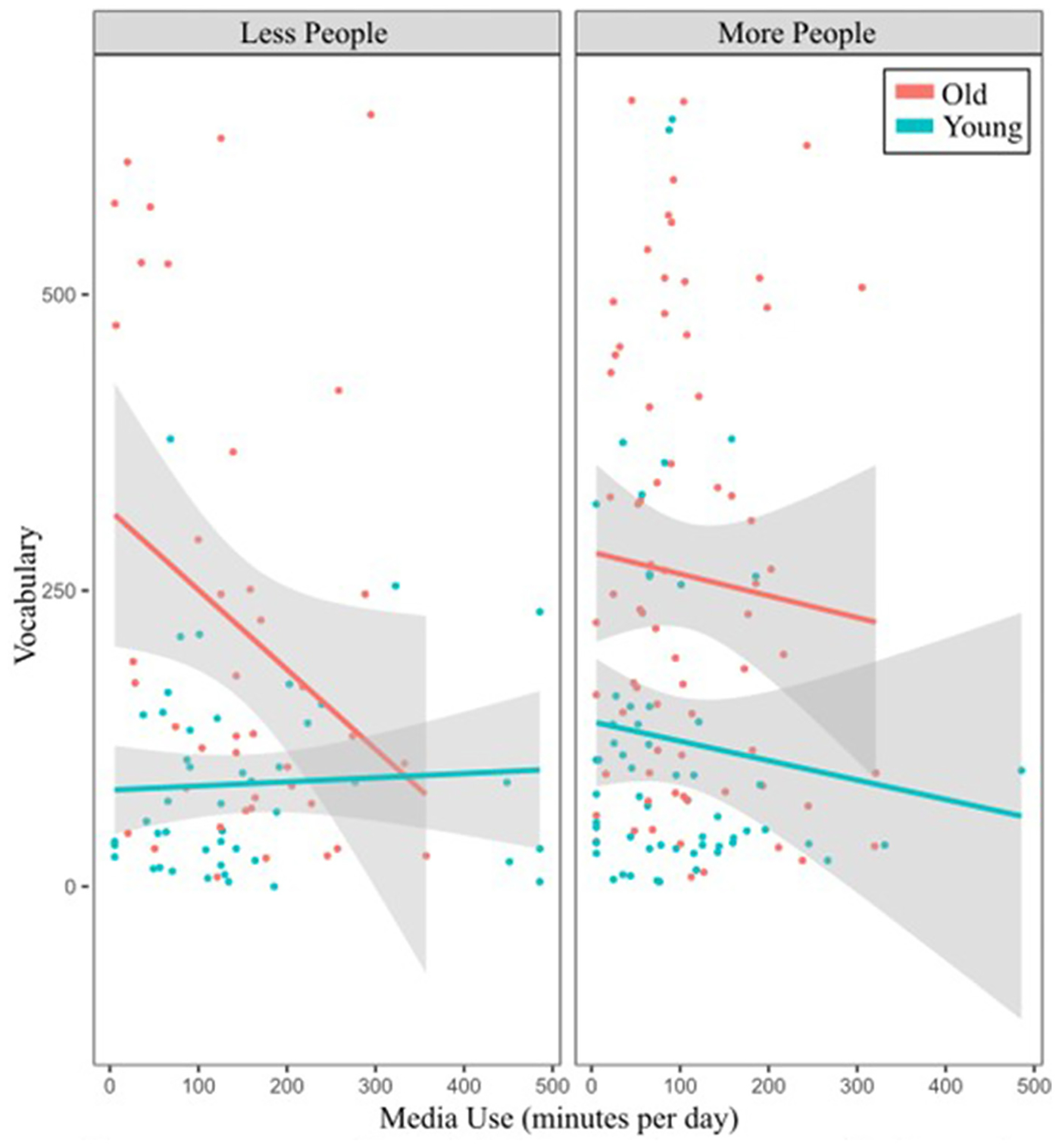
Influence of digital media use, age, and social interaction on vocabulary size. Older children (red line) and younger children (blue line) were dichotomized as older or younger than 23.48 months. Interacting with less (left plot) or more (right plot) people was dichotomized as interacting with more or less than 5.54 people.

A multiple regression model examined the interaction between age, amount of social interaction, and amount of digital media use on vocabulary, when controlling for SES (composite score). A three-way interaction between age, amount of social interaction, and amount of digital media use emerged (*b* = 36.88, *t* = 2.12, *p* = 0.04; Figure 1). To probe this three-way interaction term, we used the sim_slopes function in R (Cohen et al., 2003; Bauer and Curran, 2005). For children 19 months old and younger, there is no association between amount of social interaction, amount of digital media use, and vocabulary. For children older than 19 months old, when the number of people children interacted with was below 1.28, digital media had a negative effect on vocabulary outcome (see Supplementary Table S2 for simple slopes statistics). Among this age group, interacting with <9.81 people resulted in a negative relationship between digital media use and vocabulary, although this relationship was only marginally significant among children older than 27.94 months. These findings suggest that higher amounts of digital media use are associated with smaller vocabulary size when older children engage in fewer social interactions. This relationship is true regardless of SES. There was also a main effect of age (*b* = 90.03, *t* = 9.51, *p* < 0.001) and amount of digital media use (*b* = −21.77, *t* = −2.11, *p* = 0.04), with older children and children with lower rates of digital media use having larger vocabularies.

### 3.3 The mediating role of digital media on the relationship between SES and vocabulary

**FIGURE 2:**
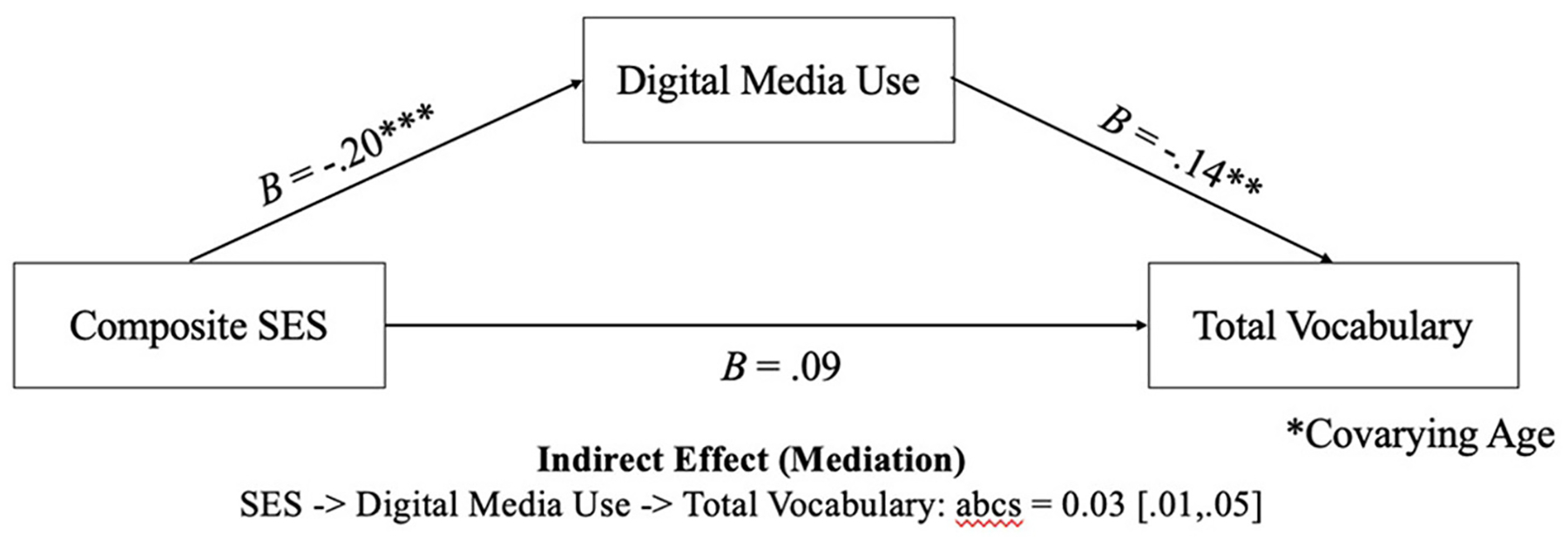
The association between SES and vocabulary was fully mediated by digital media use, when controlling for age. abcs = completely standardized indirect effect. The 95% confidence intervals for the indirect effects are contained in brackets after the point estimates and were constructed using bias-corrected bootstrapping with 5000 samples. *p < 0.05, **p < 0.01, ***p < 0.001.

We utilized mediation to identify if differences in total vocabulary knowledge related to SES could be explained by digital media use. We used the PROCESS macro (Hayes, 2022) to specify this serial mediator model with ordinary least squares path analysis (see Figure 2). To ensure age did not provide an alternative explanation for the effects of SES on the outcomes, we controlled for this variable in the serial mediation analysis. Indirect effects for the specific pathways were computed using bias-corrected bootstrapping with 5,000 samples to construct 95% confidence intervals. Intervals not containing zero indicate that the indirect effect is statistically significant. Completely standardized indirect effects were computed (labeled “abcs” in Figure 2) to obtain measures of effect size (Preacher and Hayes, 2008); values of |0.01|, |0.09|, and |0.25| are considered small, medium, and large effects, respectively. The SES-to-Digital Media Use-to-Total Vocabulary pathway emerged as significant (abcs: B = 0.03, boot S.E. = 0.01, boot 95% CI [0.01, 0.05]).

### 3.4 Can social interactions offset the relationship between SES, digital media use, and total vocabulary?

**FIGURE 3:**
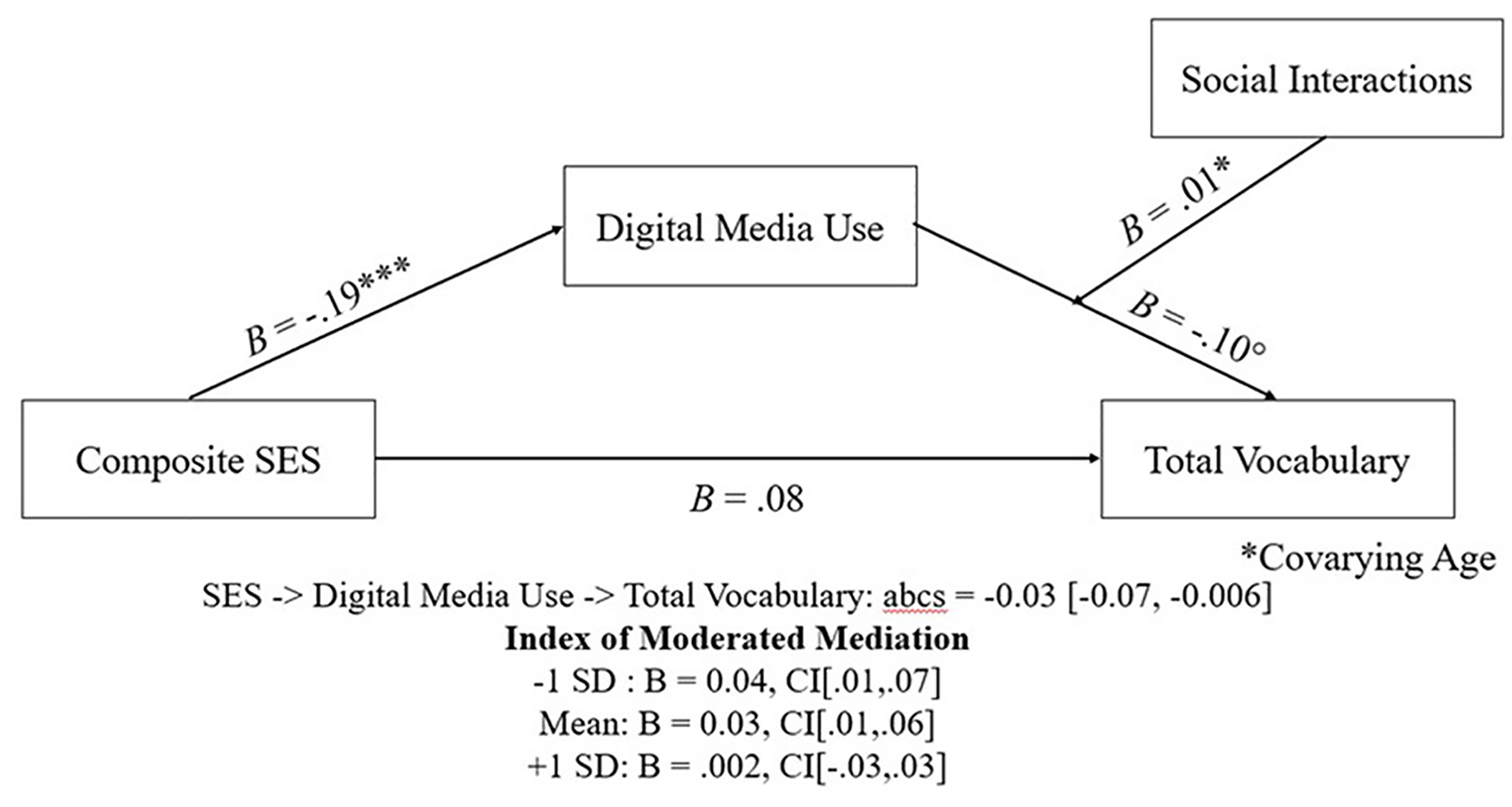
The SES to Digital Media Use to Total Vocabulary pathways varies significantly depending on the number of people a child interacts with on a regular basis. The 95% confidence intervals for the indirect effects were constructed using bias-corrected bootstrapping with 5000 samples. abcs = completely standardized indirect effect. The 95% confidence intervals for the indirect effects are contained in brackets after the point estimates and were constructed using bias-corrected bootstrapping with 5000 samples. Under the Index of Moderated Mediation, the reported mean and SDs represent the number of social interactions that significantly moderated the mediation model. ^◦^*p* < 0.10, *p < 0.05, ***p < 0.001.

Given the well-established relationship between SES and vocabulary knowledge, as well as the mediating role of digital media between these variables, we next sought to determine whether social interactions can offset this relationship. Conditional processes, also known as moderated mediation, were implemented to identify if SES-to-Digital Media Use-to-Total Vocabulary pathway was moderated by social interactions. We used the PROCESS macro (Hayes, 2022) to specify this moderated mediation model (using model 14; see Figure 3). To ensure that age did not provide an alternative explanation for the effects of social interaction on the outcome, we controlled for this variable in the analysis. Once again, bias-corrected bootstrapping with 5,000 samples was implemented to construct 95% confidence intervals for the indirect effects. Intervals not containing zero indicate that the indirect effect is regarded as statistically significant.

The SES-to-Digital Media Use-to-Total Vocabulary pathways significantly varied across SES (Index = −0.03, boot S.E. = 0.02, 95% CI [−0.07, −0.006]). The bootstrapped confidence intervals of the conditional effects indicated that individuals from lower SES households have higher amounts of digital media use, however, this only negatively impacts vocabulary if the child interacts with <5 people on a regular basis (1 SD: B = 0.04, boot S.E. = 0.02, boot 95% CI [0.01,0.08]; Mean: B = 0.03, boot S.E. = 0.01, boot 95% CI [0.01,0.06]). For children who interact with more than five people, the observed negative effects of digital media use on vocabulary are not present (+1 SD: B = 0.003, boot S.E. = 0.02, boot 95% CI [−0.03, 0.03]).

## 4 Discussion

One rising concern related to digital media use is it indirectly impacts children’s language development by removing other linguistically rich experiences such as social interactions with others. Prior work has found that more digital media use correlates with less language input and fewer conversational turns from caregivers (e.g., Sundqvist et al., 2021), however, no work has gone beyond the home environment to tap broad opportunities for social interactions in a child’s daily life. Moreover, no work has done so in conjunction with digital media use across a diverse set of families. Here, we take a first step toward such a goal and ask if media use (and specifically time spent watching videos) correlates with the overall amount of daily social interactions an individual has and how these social interactions influence children’s vocabulary knowledge. We find, consistent with other recent work, that children at this age are watching videos/TV an average of 2 h/day; a rate that has increasing risen over the past few years (Rideout and Robb, 2020; Bergmann et al., 2022). Most importantly though, 17–30-month-old children here who experience less social interaction and greater digital media use have smaller vocabularies, and this disparity widens as children develop. Moreover, children from low SES households are likely to experience greater digital media use, putting them at risk for poorer vocabulary outcomes. Importantly though, our findings suggest higher amounts of social interaction may benefit vocabulary for all children, but especially those from lower socioeconomic backgrounds.

These results also contribute to our understanding of pathways and possible mechanisms for vocabulary growth in children from a range of SES backgrounds. As has been shown in past research, children from lower SES household were more likely to have smaller expressive vocabularies than their higher SES peers. We expand on this line of work by demonstrating that a critical mediator of this relationship is digital media use, a variable which is often overlooked in studies of the vocabulary gap. This finding has important implications for caregivers and policymakers, as digital media use is a relatively malleable risk factor. In fact, several existing policies suggest caregivers should limit digital media use by children; however, despite revised recommendations by the American Academy of Pediatrics (AAP), rates of children’s digital media use continue to rise. Specifically, research has shown that from as early as 8 months of age, children have regular daily exposure to screens, which only increases with age (Bergmann et al., 2022). Digital media rates rise so much so that overall screen use among teens and tweens has increased by 17 percent from 2019 to 2021 — growing more rapidly than in the 4 years prior (Rideout and Robb, 2020). Given the increasing prevalence of digital media in children’s lives the current study suggests that increasing the number of social interactions children engage in may serve as an alternative point of intervention to offset the detrimental effects of digital media on vocabulary. This is especially true for children from lower SES backgrounds. Numerous studies have cited the importance of social interactions in children’s language development (Weisleder and Fernald, 2013; Tamis-LeMonda et al., 2014; Romeo et al., 2018). However, only a few studies have highlighted the importance of considering “other” social partners, beyond primary caregivers, in capturing the language input provided to children (Sperry et al., 2019). The current findings demonstrate that by interacting with more social partners, low SES children are more likely to experience gains in vocabulary that would otherwise be negatively influence by digital media use.

The current work highlights the moderating effect broader social interactions may have on digital media’s impact on language development. Children who engaged in higher rates of digital media use were less likely to interact with more people and had smaller vocabularies, replicating past findings that digital media use decreases opportunities that are critical for language development (Christakis et al., 2009; Anderson and Hanson, 2017; Sundqvist et al., 2021). Here, only the overall amount of social interactions was measured via a single item on a parent report survey, which could include everything from playing with siblings, to talking with grandparents, to interacting with a classroom of preschoolers. Despite the variability in source of the social interactions, the effect of simply being around more people seems to offset the negative impacts of digital media use on vocabulary. This is consistent with prior work showing that interactions with both caregivers (Ramírez-Esparza et al., 2014) and peers (Perry et al., 2018) can boost language, primarily because it leads to more opportunities for using and learning new words. Future work ought to further test these mechanistic explanations with extended recording of both media use and social interactions outside the home. Differentiating social interaction inside and outside the home may also prove to be a fruitful endeavor as various lines of work suggest that household routines and chaos could factor into children’s language (e.g., Lecheile et al., 2020). Moreover, the current study does not incorporate social interactions *during* digital media use which may be even more beneficial for language – prior work finds digital media use that is interactive, social, and used with a social partner is more facilitative to language learning (Linebarger and Vaala, 2010; Lytle et al., 2018). It is also possible that the types of words and quality of word learning experienced in social interactions differs from those in digital interactions – another avenue for future work.

Taken together, these results suggest that the growing prevalence of digital media in young children’s daily lives may have negative impacts on vocabulary, but critically, social interactions alter that impact in a positive way, possibly by providing opportunities for hearing and using language (Sundqvist et al., 2021). By increasing attention to the overall daily social interactions of young children we may be able to offer ways to mitigate media’s negative effect on children’s early vocabulary growth, across all socioeconomic backgrounds.

## Supplementary Material

Supplement

## Figures and Tables

**TABLE 1:** Pearson’s R Correlations between demographic and behavioral variables.

	1	2	3	4	5	6	8	9
(1) Age in days	1							
(2) Vocabulary	0.47***							
(3) Maternal education	0.04	0.13*	1					
(4) Paternal education	0.12	0.12*	0.59***	1				
(5) Average parent education	0.08	0.14*	0.89***	0.9***	1			
(6) Income	0.05	0.13*	0.47***	0.4***	0.49***	1		
(8) Social interaction	0.12*	0.06	0.07	0.04	0.06	0.15*	1	
(9) Digital media use	0.06	−0.13*	−0.21***	−0.16**	−0.21***	−0.17**	−0.21***	1

* *p* < 0.05,

** *p* < 0.01,

*** *p* < 0.001.

## Data Availability

The original contributions presented in the study are publicly available. This data can be found here: https://osf.io/mv2ua/?view_only=2793f7f362824eafb81d0d5d43ea9608.

## Footnotes

1 Though just a single item was used to measure social interaction here, this particular question demonstrated strong internal validity with unpublished variables from this same data set. For instance, the average number of social interactions incrementally rises along with the number of people involved in a child’s childcare situation - children’s whose primary source of childcare is a parent, nanny, or other close relative interact with an average of 4.6 people/day, whereas those who report childcare that is primarily in a larger group setting (>5 children) average 13.1 people/day.
