# Supplementary material for: Social interactions offset the detrimental effects of digital media use on children’s vocabulary
Source: Front Dev Psychol. Author manuscript; Available in PMC 2024 Jun 28. (PMC11213284; doi:10.3389/fdpys.2024.1401736)
Supplement: Supplement [file NIHMS2004475-supplement-Supplement.docx]

**Supplemental Materials**

Social interactions offset the detrimental effects of digital media use on children’s vocabulary

*Table S1.*Sample demographic information.

|  |  | **Parent n** | **Child n** |
| --- | --- | --- | --- |
| **Gender** | Female | 209 | 135 |
|  | Male | 94 | 168 |
|  | Non-binary/Not Reported | 2 | 1 |
| **Race** | White | 248 | 232 |
|  | Black | 33 | 30 |
|  | Asian | 8 | 7 |
|  | American Indian | 2 | 3 |
|  | Mixed Race | 8 | 30 |
|  | Not Listed | 6 | 3 |
| **Ethnicity** | Hispanic | 23 | 34 |
| **Employment Status** | Not employed | 67 |  |
|  | One part-time job | 36 |  |
|  | One full-time job | 182 |  |
|  | Multiple jobs | 13 |  |
|  | Parental leave | 5 |  |
|  | No Response | 2 |  |
| **Socioeconomic status info** | Education level | 5.2 (1.3) |  |
|  | Income | $75,000 ($36,000) |  |

Note: Mean SES information presented with SD in parenthesis. Parent education is the average of caregivers rank ordered with 1 as <7^th^ grade education and 8 as doctoral degree

Table S2. Simple slopes

| **Age in Months** | **Number of People** | **Estimate** | **Standard Error** | **t-value** | **p-value** |
| --- | --- | --- | --- | --- | --- |
| -1 SD (<19.03) | -1 SD  (<1.28) | 2.63 | 20.33 | 0.13 | 0.90 |
|  | Mean  (1.28-9.81) | -16.69 | 14.85 | -1.12 | 0.26 |
|  | +1 SD (>9.81) | -36.01 | 33.40 | -1.08 | 0.28 |
| Mean  (19.03-27.94) | -1 SD  (<1.28) | -41.91 | 12.57 | -3.33 | 0.00** |
|  | Mean  (1.28-9.81) | -21.34 | 10.18 | -2.10 | 0.04* |
|  | +1 SD (>9.81) | -0.78 | 19.99 | -0.04 | 0.97 |
| +1 SD  (>27.94) | -1 SD  (<1.28) | -86.45 | 20.18 | -4.28 | 0.00** |
|  | Mean  (1.28-9.81) | -26.00 | 14.53 | -1.79 | 0.07° |
|  | +1 SD (>9.81) | 34.46 | 28.45 | 1.21 | 0.23 |

Note: Simple slopes analysis probing three-way interaction between age, social interaction, and digital media use on vocabulary size. Estimates represent the slope of the line between digital media use and vocabulary size at each interval of age and number of social interactions. °*p* < 0.10, **p* < 0.05, ***p <* 0.01.
